# Editorial: Mechanism and therapeutic potential of macrophages in metabolic and cardiovascular diseases

**DOI:** 10.3389/fimmu.2023.1352567

**Published:** 2023-12-19

**Authors:** Xuqiang Nie, Youcai Deng, Xiaoyong Tong, Felicity Han

**Affiliations:** ^1^ College of Pharmacy, Zunyi Medical University, Guizhou, China; ^2^ Joint International Research Laboratory of Ethnomedicine of Ministry of Education & Key Laboratory of Basic Pharmacology of Ministry of Education, Zunyi Medical University, Guizhou, China; ^3^ Department of Clinical Hematology, College of Pharmacy and Laboratory Medicine Science, Army Medical University, Chongqing, China; ^4^ School of Pharmaceutical Sciences, Chongqing University, Chongqing, China; ^5^ Australian Institute for Bioengineering and Nanotechnology, The University of Queensland, Brisbane, QLD, Australia

**Keywords:** macrophages, cardiovascular diseases, atherosclerosis, metabolic diseases, therapeutic potential, diabetic nephropathy, GBP2

Macrophages have a crucial role in the pathogenesis and progression of metabolic and cardiovascular diseases, including diabetes, hypertension, atherosclerosis, and heart disease (1–3). It is important to understand the mechanisms underlying their involvement and explore their therapeutic potential to develop effective treatments for these conditions. In the context of metabolic disorders, such as obesity and insulin resistance, macrophages play a significant role (4). These immune cells infiltrate adipose tissue and contribute to chronic inflammation by releasing pro-inflammatory cytokines. This inflammatory environment disrupts insulin signaling pathways, leading to impaired glucose metabolism and an increased risk of diabetes.

Furthermore, macrophages are involved in the formation and progression of atherosclerosis, a major cardiovascular disease. When cholesterol accumulates within arterial walls, macrophages migrate to these sites and engulf oxidized low-density lipoproteins (LDL) (5). However, excessive uptake leads to the formation of foam cells, which are a characteristic feature of early-stage atherosclerotic lesions.

Moreover, activated macrophages release various enzymes that degrade extracellular matrix components within plaques (6). This process weakens plaque stability and increases the likelihood of rupture or erosion. Additionally, the cytokines released by macrophages attract other immune cells, such as T lymphocytes, into plaques, further exacerbating inflammation.

The objective of our Research Topic is to investigate and disseminate studies that have emphasized the potential therapeutic applications of macrophage-targeted treatments for various inflammatory diseases. Li et al. investigated the identification of key genes associated with diabetic nephropathy (DN) and the pro-inflammatory M1 macrophage phenotype. The study identified four hub genes, namely *CASP1*, *MS4A4A*, *CD53*, and *GBP2*, which are involved in the progression of DN through the pro-inflammatory M1 macrophage phenotype. Among these genes, *GBP2* showed promise as a prognostic biomarker and potential intervention target for DN by regulating M1 polarization. Moreover, the study revealed that GBP2 activates the Notch1 signaling pathway to drive M1 polarization of macrophages. The article also discussed the potential of GBP2 as a candidate target for mitigating renal tubular injury, emphasizing the need for further basic experiments and clinical trials to fully comprehend the role of GBP2 in the context of DN.

Lisk et al. investigated the effects of moderate hypoxia on circulating monocytes and tissue resident macrophages in Berkeley sickle cell anemia mice. The study found that the metabolism of these cells, isolated from the lung, spleen, and peripheral blood, differed between those exposed to moderate hypobaric hypoxia and those in normoxic conditions. Moreover, cardiovascular phenotypes also varied between wild type and Berk mice exposed to moderate hypoxia. Berk mice exhibited higher right ventricular systolic pressures, a lower ventricular to vascular coupling ratio, and a higher Fulton index. Additionally, the metabolic phenotypes of peripheral blood mononuclear cells (PBMCs), spleen, and lung macrophages showed significant differences between wild type and Berk mice. Notably, each cell type displayed distinct metabolic responses to moderate hypoxia. These findings offer valuable insights into the reprogramming of energy metabolism and altered mitochondrial metabolism in response to hypoxia in the context of sickle cell anemia.

Zhang et al. examine the crucial role of mitophagy in atherosclerosis (AS) and its potential as a therapeutic target. Mitophagy, a form of autophagy, selectively removes damaged and depolarized mitochondria to maintain cellular homeostasis. The paper emphasizes the significance of mitophagy in the homeostasis and physiological function of vascular endothelial cells, vascular smooth muscle cells, and macrophages, which are all essential in AS development. It explores the mechanisms and therapeutic advancements of mitophagy in different cell types, shedding light on the impact of factors such as ROS, glucose and lipid metabolism disorders, and hypoxia on mitophagy dysfunction. The findings suggest that malfunctioning mitophagy may result in endothelial cell damage, proliferation and phenotypic switching of vascular smooth muscle cells, altered polarization of macrophages, and potential cell death, all contributing to AS development. Furthermore, the review highlights the potential of medications and natural chemicals to modulate mitophagy and decelerate the progression of AS, providing valuable insights for potential AS management strategies.

Wu et al. conducted a review on the role of macrophage polarization in the progression of atherosclerosis and the emerging therapies for its regulation. Atherosclerosis is a chronic inflammatory condition that primarily affects large and medium arteries. Macrophages play a crucial role in all stages of atherosclerosis development and progression. The review suggests that controlling the polarization of macrophages could effectively control the progression of atherosclerosis, making it an important target for therapeutic interventions. The review emphasizes the significance of targeting macrophage polarization for the prevention and treatment of atherosclerosis, as lipid-lowering therapy alone may not completely delay its progression. It also highlights the potential of regulating macrophage polarization and promoting phenotypic transformation to anti-inflammatory subtypes as an effective approach. The distribution of macrophage subtypes in atherosclerotic plaques and the potential of single-cell analysis technology for further elucidation are also discussed. The review concludes by emphasizing the need for further *in vivo* and clinical studies to understand the modulation of macrophage polarization as an effective approach for atherosclerosis treatment, presenting it as a compelling avenue for future research.

Further research is needed to fully elucidate the complex mechanisms by which macrophages contribute to metabolic and cardiovascular diseases. Identifying specific molecular targets involved in macrophage activation may lead to more targeted therapies with fewer side effects. Additionally, conducting clinical trials to evaluate the efficacy of interventions targeting macrophages is necessary for translating these findings into clinical practice.

In conclusion, understanding the mechanisms underlying macrophage involvement in metabolic and cardiovascular diseases provides valuable insights into disease pathogenesis and offers opportunities for therapeutic intervention. By targeting macrophage function or modulating their polarization state, it may be possible to develop novel treatments aimed at reducing chronic inflammation and improving outcomes for patients affected by these conditions.

## Author contributions

XN: Writing – original draft, Writing – review & editing. YD: Writing – review & editing. XT: Writing – review & editing. FH: Writing – review & editing.
